# Recent advances on coxsackievirus A6 vaccine research

**DOI:** 10.3389/fimmu.2025.1603028

**Published:** 2025-06-06

**Authors:** Xianfeng Zhou, Han Mo, Hui Li, Fenglan He, Qian Yang

**Affiliations:** ^1^ Cancer Research Center, Jiangxi University of Chinese Medicine, Nanchang, China; ^2^ Jiangxi Provincial Health Commission Key Laboratory of Pathogenic Diagnosis and Genomics of Emerging Infectious Diseases, Nanchang Center for Disease Control and Prevention, Nanchang, China; ^3^ National Key Laboratory of Intelligent Tracking and Forecasting for Infectious Diseases (NITFID), National Institute for Viral Disease Control and Prevention, Chinese Center for Disease Control and Prevention, Beijing, China; ^4^ National Polio Laboratory, World Health Organization Polio Reference Laboratory for the Western Pacific Region, National Institute for Viral Disease Control and Prevention, Chinese Center for Disease Control and Prevention, Beijing, China; ^5^ National Health Commission Key Laboratory of Laboratory Biosafety, National Institute for Viral Disease Control and Prevention, Chinese Center for Disease Control and Prevention, Beijing, China

**Keywords:** HFMD, herpangina, coxsackievirus A6, genetic recombination, vaccine, preclinical evaluation

## Abstract

**Background:**

Hand, foot, and mouth disease (HFMD) is an acute infectious disease caused by human enteroviruses (EVs). EVs are most prevalent in children under five years of age and have the potential to result in herpangina, HFMD, and severe complications, including encephalitis and death. Since the first outbreak was reported in 2008 in Finland, coxsackievirus A6 (CVA6) has spread rapidly and frequently undergone recombination events worldwide, posing a threat to the health of pediatric population around the globe.

**Aim of review:**

The dearth of vaccines and anti-CVA6 drugs hinders the efficient prevention and control of CVA6. However, over the course of the last decade, researchers have endeavored to develop potential vaccine candidates for CVA6 using various pathways. In this study, we present a systematic review of research progress pertaining to the CVA6 vaccines, with a particular emphasis on the most recent advancements in CVA6 vaccine development and evaluation. The objective of this review is to establish a theoretical foundation for the formulation of preventive and control strategies, as well as the development of vaccines against not only CVA6 but also other key serotypes in the future.

**Key scientific concepts of review:**

The review comprehensively addresses the diverse array of CVA6 vaccine development, encompassing a range of modalities such as inactivated, virus-like particle, and subunit vaccines, among others. A systematic analysis was conducted on animal-based assessments of various CVA6 vaccines, encompassing immunogenicity, protection rate, and cross-immunization as critical evaluation parameters. In light of the recurrent recombination of CVA6 and the evolution of pathogen profiles, the recommendation is made for the future development of multivalent and mRNA vaccines, which hold significant potential in the prevention and control of CVA6 and other major dominant serotypes.

## Graphical Abstract

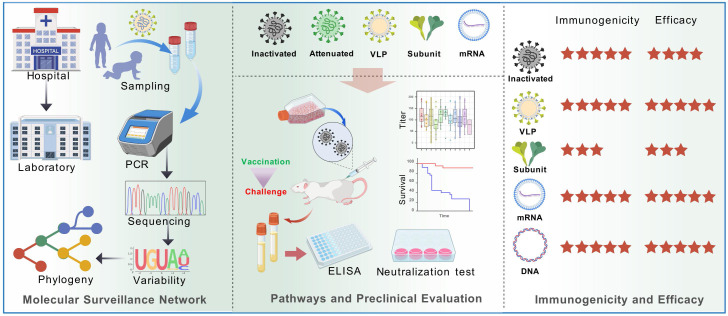

## 1 Introduction

Hand, foot and mouth disease (HFMD) is a prevalent infectious disease that predominantly afflicts young children five years of age or younger (1). HFMD is caused by human enteroviruses (EVs) and is characterized by the presence of fever and herpes-like ulcers on the hands, feet, mouth and buttocks (2). While the majority of cases are self-limiting and mild, some cases may rapidly progress to severe neurological and systemic complications, including aseptic meningitis, encephalitis and myocarditis (3). EVs can be categorized into four species: EV-A, EV-B, EV-C and EV-D (4). The EV-A includes coxsackievirus group A (CVA) and various enteroviruses, which are responsible for over 90% of HFMD cases, with the particular strain known as EV-A71. EV-A71 and CVA16 were once regarded as major pathogens. Nevertheless, since the first reported CVA6 outbreak occurred in Finland in 2008, epidemiological surveillance data on a global scale have demonstrated a substantial shift of pathogen spectrum (5). Subsequent surveillance in many countries and regions has shown a consistent rise of CVA6, and it has become a globally dominant pathogen of HFMD and herpangina (6–8). The EV-A71-associated HFMD was responsible for a significant number of fatalities in China between 2008 and 2012 (9), thus prompting the development of EVA71 vaccines around 2015 (10). Following the extensive utilization of the vaccine in China, a substantial decline in EV-A71 prevalence has been observed, accompanied by a notable alteration characterized by the domination of CVA6 (10–12).

**Figure 1:**
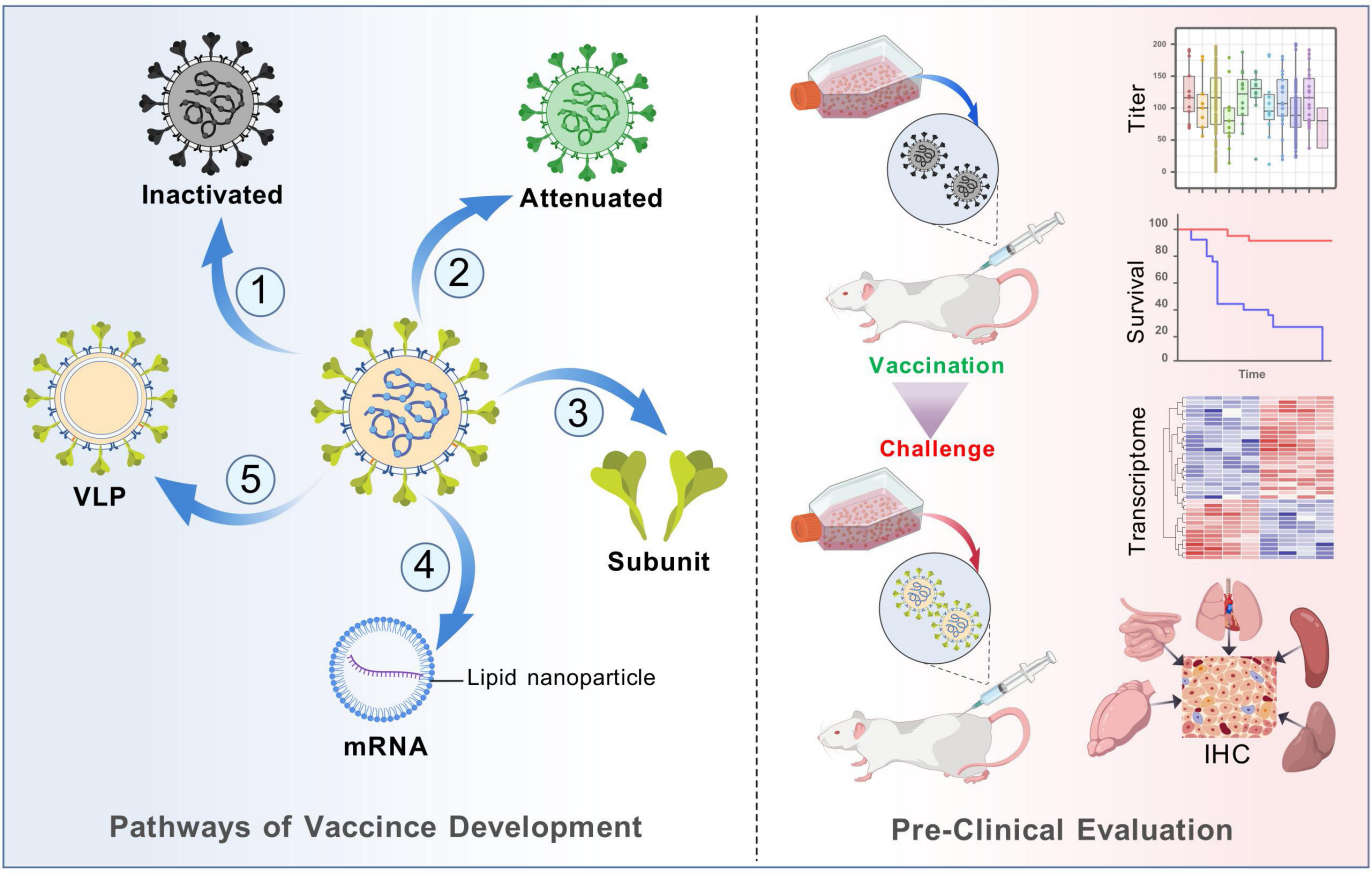
Diagram of main pathways of viral vaccine development and mouse model-based pre-clinical evaluation. Multiple technology pathways of viral vaccines (not limited to the diagram) play a key role in eradication of infectious diseases such as smallpox and polio. Vaccine assessment is a crucial process to evaluate the efficiency and safety. Cell culture provides strain isolation and clone, while animal models serve for evaluation of pathogenicity, immune response and protection.

CVA6 has been demonstrated to result in a greater extent of skin lesions and more severe tissue destruction. For instance, it has been shown to cause atypical clinical manifestations such as rashes outside of the typical lesions, flaking of the palms and soles of the feet, and nail loss (13–15). These symptoms can be accompanied by flu-like symptoms and higher fever, which can last longer than typical non-CVA6 HFMD (16). At present, there is an absence of specified treatment and prevention, with management relying on non-pharmacological interventions (NPIs) (17). Despite the advanced state of viral vaccine development (**Figure 1**), the development of a CVA6 vaccine currently faces a number of challenges: 1) screening of candidate strains under conditions of frequent recombination events; 2) efficient and safe development of vaccine pathways; 3) conflict between long-term vaccine research and the rapid shift of pathogen spectrum (18); 4) The accumulation of mutations in epitopes has the potential to result in antigenic drift, a process that can diminish the immunoprotective efficacy of the vaccine. Therefore, in order to perform a comprehensive evaluation of CVA6 immune evasion, molecular surveillance, epitope selection pressure analysis, and serological investigation are necessary. Consequently, the development of efficient multivalent vaccines has become a pressing necessity for the prevention and management of HFMD. This review aims to analyze existing vaccine development strategies and preclinical evaluation approaches, with a view to providing valuable strategies for the development of efficient enterovirus vaccines, particularly multivalent vaccines.

## 2 Biological characteristics of CVA6

**Figure 2:**
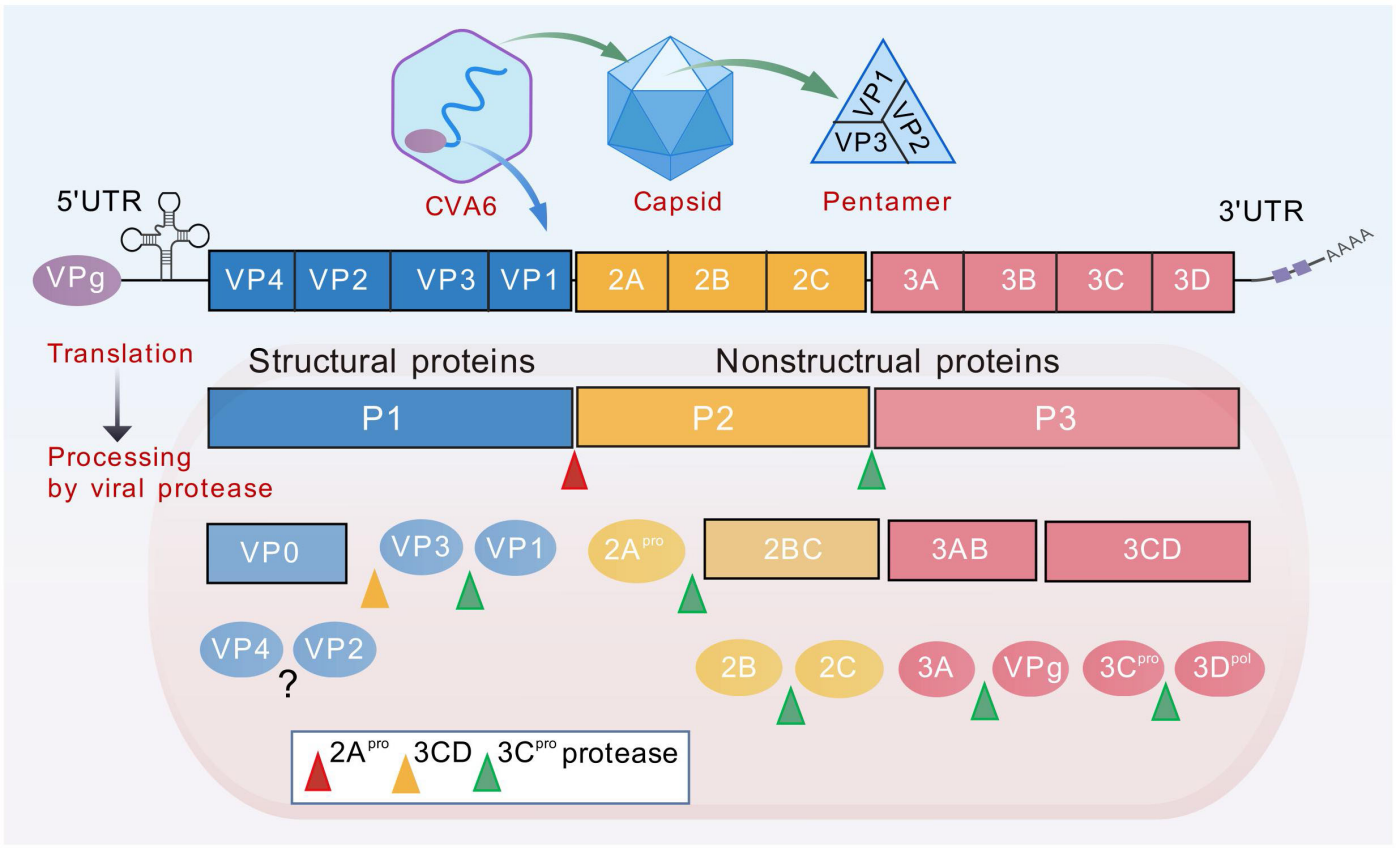
Diagram of CVA6 genome structure. The genome (~7400bp) is translated to produce polyproteins P1-P3. The P1 region encodes four structural proteins VP1-VP4, while the P2/P3 region encodes seven non-structural proteins, including 2A-2C and 3A-3D. The viral genome is covalently linked to the viral protein VPg (3B), which is required as a primer for replication. Genome translation yields a single polyprotein, which is subsequently proteolytically cleaved into two distinct groups of proteins. The first group consists of the replication proteins (2A–2C and 3A–3D), while the second group comprises the capsid proteins (VP0, VP1, and VP3). The maturation process of the virus involves the synthesis of three structural proteins (VP0, VP1, VP3) in the P1 region of the CVA6 virus, followed by the cleavage of VP0 into VP2 and VP4 to form a complete capsid protein. The process of genome replication by the viral RNA-dependent RNA polymerase (3D^po^l) commences with the synthesis of a negative-strand (–) RNA molecule that functions as a template for the subsequent synthesis of new (+) RNA molecules. Replication occurs within membranous replication organelles, where a conducive lipid environment is established by viral proteins 2BC and 3A.

CVA6 belongs to the species EV-A, genus *Enterovirus*, in the family *Picornaviridae (* 19, 20). It is a single-stranded, positive-sense, non-enveloped RNA virus with a genome of approximately 7,400 nucleotides (**Figure 2**) (5). Following entry into the host cell, the genome is translated to produce polyproteins. The P1 region is responsible for encoding four structural proteins (VP1-VP4), while the P2/P3 region encodes seven non-structural proteins, including 2A-2C and 3A-3D (21). The capsid structure consists of four subunits: VP1, VP2, VP3 and VP4.The process of viral adsorption, infection and immune escape is mediated by the spatial conformation formed by the exposed VP1-VP3 proteins on the surface of the capsid (**Figure 2**). The process of viral assembly and infection is facilitated by the VP4 protein attached to the inner surface of the capsid, and the hydrophobic pocket below the bottom of the protein canyon usually contains lipid pocket factors, which VP1 uses to stabilize the viral particle (20). Furthermore, the VP1 pocket in the CVA6 VLP was found to be devoid of any protein, a factor which has been demonstrated to have a negative effect on the stability of the particles (22).

As indicated by the extant literature, the identification of divergent EV serotypes is predicated on the analysis of the VP1 sequence (23). The VP1 surface loop structure has been shown to be the key antigenic epitope (24). It has been determined that the most significant structural differences occur in the CVA6, CVA16, and EVA71 four surface loops (BC, DE, EF, and HI), and that these differences in the structure of key antigenic epitopes of different serotypes may be an important mechanism for the low cross-protection rate of existing vaccines (24, 25).

## 3 Evolutionary feature and recombination events of CVA6

CVA6 was first isolated in the United States in 1949, and the first CVA6 outbreaks was recorded in Finland in 2008 (5). CVA6 have become a predominant agent globally, particularly in Asia-Pacific (17, 26–28) and Southeast Asia (29–32), and Europe (8, 33). CVA6 mainly affects children under 5 years of age, and is more likely to infect adults than other serotypes (34).

**Figure 3:**
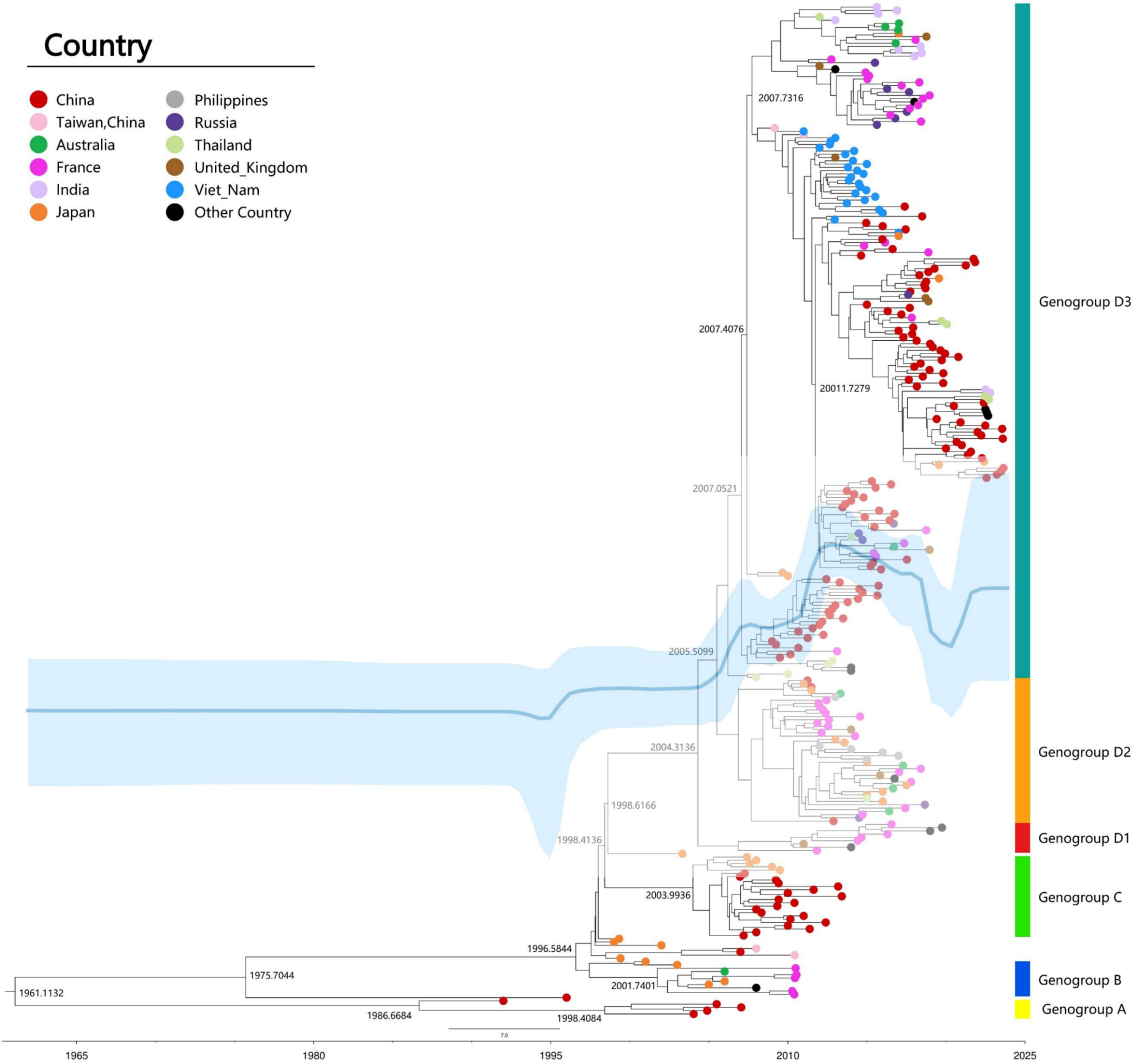
Maximum clade credibility (MCC) tree of 309 representative VP1 sequences (915bp) of CVA6 strains worldwide. MCC tree was constructed using TreeAnnotator, and the burn-in option was used to remove the first 10% of the sampled trees; the resulting tree was visualized using FigTree (v1.4.4). The Markov chain Monte Carlo (MCMC) method implemented in BEAST (v1.8.4) was used to estimate the divergence time, temporal phylogenies and rates of evolution. Blue line: Bayesian skyline plots of viral relative genetic diversity; The light blue shadow: 95% CI.

The classification of CVA6 is determined by VP1 genotyping, which reveals four distinct genotypes (A-D). Genotype A serves as the prototype (Gdula), while genotypes B, C, and D are subdivided into respective sub-genotypes (B1-B2, C1-C2, and D1-D3) (**Figure 3**). The D3 subtype can be further subdivided into sub-branches D3a, D3b, and so on (6, 27, 35–37). Clinical samples collected from different geographic regions of China indicated that the evolution of strains has been characterized by distinct phases: D2 CVA6 was predominant before 2009, genotypes D2 and D3 co-circulated from 2009-2012, and the majority of the CVA6 strains after the outbreak in 2013 belonged to the D3a branch (35). The most recent common ancestor (tMRCA) of the D3 subtype in China was dated to 2005, which preceded its initial identification in 2011, thereby indicating that this subtype had already been implicitly transmitted prior to the outbreak. Phylogenetic analysis revealed that the D3 subtype in China exhibited a close relation to strains isolated from Vietnam, Thailand, and France, suggesting widespread cross-regional transmission (**Figure 3**) (10, 38).

CVA6 recombination, linked to pathogenicity (39, 40), primarily occurs in non-structural regions (2A-2C, 3D, 5′-UTR), facilitating viral evolution (41). Analysis of 1032 genomes identified 24 recombinant forms (RF-A to RF-X) (6). Shifts in predominant strains, such as D3/A to D3/H and D3/N in France (8), and D3-Y in India (32), suggest recombination drives epidemiological changes. Enhanced viral diversity complicates vaccine development and outbreak control, necessitating continuous molecular surveillance to guide effective prevention strategies.

## 4 CVA6 vaccine research and development

**Figure 4:**
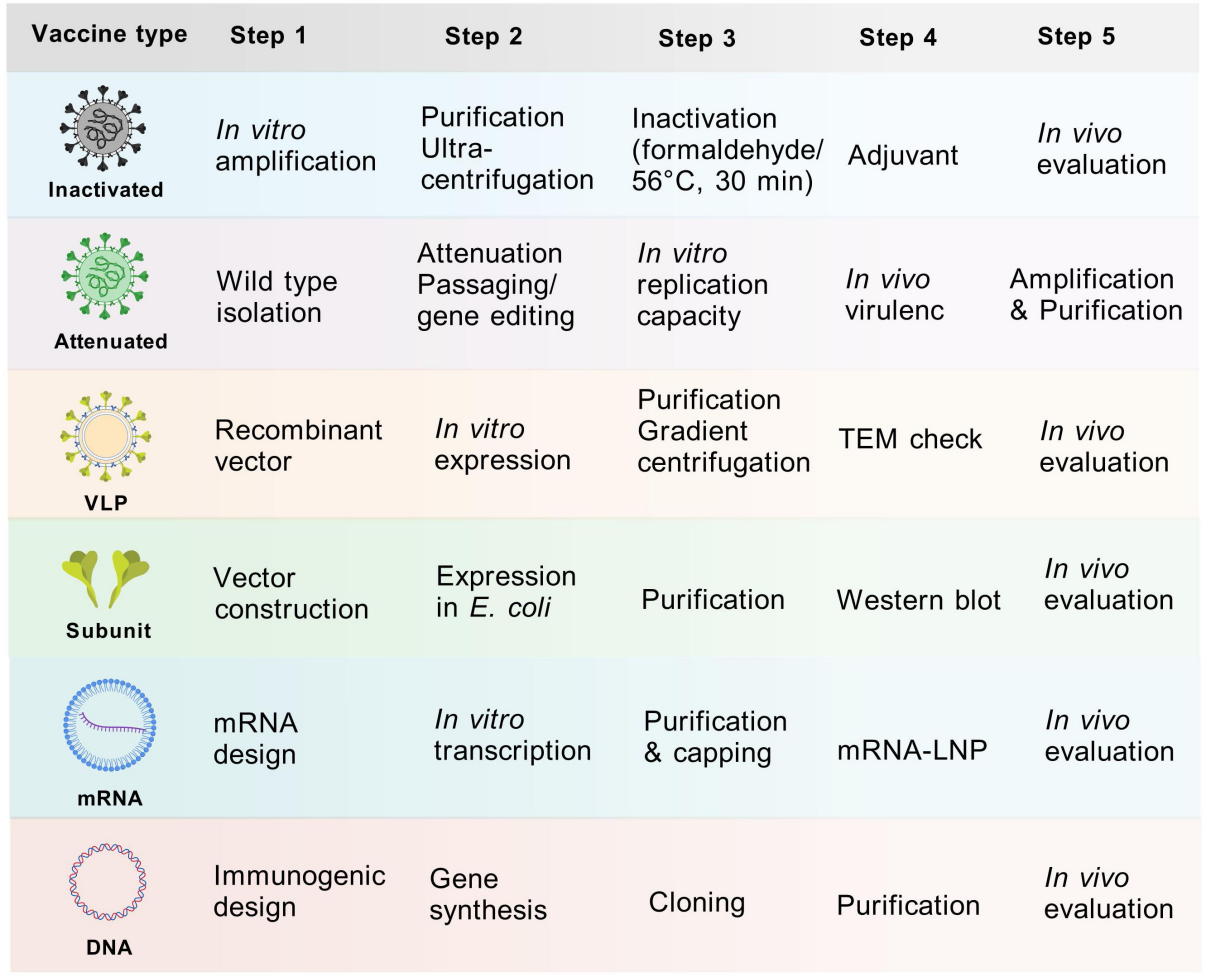
Vaccine development process of different vaccine platforms. Major steps from vaccine design to *in vivo* evaluation were displayed. The purification process entails the use of sucrose gradient centrifugation, while the observation of structure involves the utilization of electron microscopy. The inactivation process was predominantly achieved through the use of formaldehyde or 56°C for 30 minutes.

Since 2015, the inactivated monovalent EV-A71 vaccines based on C4a subtypes have been available (42). The vaccine was the first EV vaccine to be approved by the China FDA, and it demonstrated non-toxicity, high safety, and immunogenicity in clinical investigations (43, 44). However, the inactivated EV-A71 vaccine did not cross-protect against other serotypes, leading to a shift in the pathogen spectrum after vaccination (45). This phenomenon underscores the necessity for the development of multivalent vaccines. Despite the unavailability of CVA6 vaccine, approximately 20 studies have been dedicated to its development globally over the past decade (**Table 1**). Here, we present and discuss in detail the progress of CVA6 vaccine research with different vaccine platforms (**Figure 4**).

**Table 1:** Progress of CVA6 vaccine development worldwide, 2015-2025.

Nation	Year	Vaccine type	Serotype(s)	Inactivation	Purification	Structural characterization	Ref.
China	2025	VLP, mRNA	CVA6	NA	VLP: Sucrose Gradient Centrifugation mRNA: RNeasy Mini Purification	Transmission electron microscopy (TEM)	(46)
China	2025	VLP	CVA6	NA	linear 15–45% sucrose gradient at 153,900 g for 4.5 h at 4°C	NA	(47)
China	2024	VLP	CVA6	NA	Sucrose Gradient Centrifugation	SDS-PAGE, TEM	(48)
Thailand	2024	DNA	CVA6, CVA10, CVA16, EV-A71	NA	NA	NA	(49)
China	2022	Inactivated	CVA6	56°C for 30 min	NA	NA	(50)
China	2021	Inactivated	CVA6	Formaldehyde inactivation	NA	NA	(51)
China	2021	Inactivated	CVA6	56°C for 30 min	NA	NA	(52)
China	2020	Sub-unit	CVA6, CVA10, CVA16, CVB3, EV-A71	NA	NA	NA	(53)
China	2018	VLP	CVA6, CVA10, CVA16, EV-A71	NA	10–50% sucrose-gradient ultracentrifugation	TEM	(54)
Korea	2018	Inactivated	CVA6, CVA10, CVA16	1. formaldehyde at 37°C for 5 days;2. 0.025% BPL at 4°C for 3 days.	Ultracentrifugation at 25,000 rpm	NA	(55)
China	2018	Inactivated	CVA6, CVA10	Formaldehyde	Sucrose Gradient Centrifugation	TEM and SDS-PAGE	(56)
China	2017	Inactivated	CVA6	Formaldehyde	NA	NA	(57)
China	2016	Inactivated	CVA6, CVA10, CVA16, EV-A71	Formaldehyde	sucrose gradient ultracentrifugation	SDS-PAGE	(58)
China	2016	VLP	CVA6	NA	Sucrose Gradient Centrifugation	TEM	(59)
China	2016	VLP	CVA6	NA	NA	NA	(60)
China	2016	Inactivated	CVA6	Formaldehyde	NA	NA	(61)
USA	2015	Inactivated	CVA6, CVA16, EV-A71	Ethyleneimine	Sucrose Gradient Centrifugation	NA	(62)

NA, Not available.

### 4.1 Inactivated vaccines

Inactivated vaccines constitute a substantial proportion of viral vaccine development, owing to their numerous advantages, including a more mature production process, strong immunogenicity and long-lasting protective efficacy. Notable vaccine candidate cells include African green monkey kidney cells (Vero) and human embryonic lung diploid fibroblasts (MRC-5, KMB17) (63). However, the development of an inactivated vaccine is currently hindered by critical bottlenecks: 1) CVA6 proliferates inefficiently in conventional vaccine-producing cell lines Vero and MRC-5; 2) human rhabdomyosarcoma (RD) cells, which can be used for CVA6 isolation, do not meet the criteria for vaccine production as its tumorigenic risk. Consequently, there are two primary avenues for enhancing cultured cell lines: 1) enhancing the yield of conventional vaccine-producing cell lines; 2) developing new cell lines with high yield that meet the vaccine production standards. Zhang et al. utilized the principle of key cell receptor KREMEN1 (KRM1) for CVA6 infection, and constructed the Vero cell line Vero-KRM1_#1 with overexpressed KRM1 to promote CVA6 infection using isolated strain CVA6-TW-00141 (GenBank accession no. KR706309, D1 subtype) and infectious clones prepared from CVA6-HLJ11 stain (GenBank accession no. MN845762, D2 subtype), CVA6-YN17 strain (GenBank accession no. MN845882, D3 subtype) and CVA6-GD13 strain (GenBank accession no. KF682363, D3 subtype) (47). The study revealed that Vero-KRM1_#11 cells, which express elevated levels of KRM1, exhibited a substantial augmentation in the infection efficiency of CVA6 strains. These cells demonstrated a growth rate that was analogous to that of the wild-type Vero cells. Furthermore, the KRM1 expression level exhibited stability following successive passages, a property that facilitated the acquisition of high-titer virus batches through consecutive passages, thereby addressing the requirements for vaccine production. Although the modification of Vero cell lines to express the KRM1 receptor provides new ideas and tools for the development of CVA6 vaccines, genetic modifications that lead to the overexpression of receptors may have regulatory implications. Therefore, the application of receptor overexpressing modified cell lines in human vaccine production necessitates further validation in terms of safety, stability, and other factors.

To date, *in vivo* results have indicated that the inactivated vaccines have induced immune responses and demonstrated different levels of protection in different laboratories (**Supplementary Table 1**). For instance, a Korean study of an inactivated trivalent vaccine found significant differences in immune protection with CVA6, CVA10 and CVA16 challenge in BALB/c (55). A recent study evaluated the efficacy of a coxsackievirus A6 vaccine candidate in an actively immunized mouse model, in which 1.5 and 4.5 µg of the inactivated CVA6 vaccine were used to challenge two clones (CVA6-R5 and CVA6-R10) of different virulence. The mice were completely protected from death in 14 days (51).

Previous studies indicated that the immunogenicity of inactivated vaccines produced by different inactivation methods may differ (**Supplementary Table 1**). Qian et al. conducted a comparative analysis of the immunogenicity of a trivalent vaccine (CVA6, CVA10 and CVA16) and two types of inactivators, formaldehyde and β-propiolactone (BPL) (51). The results demonstrated that the two inactivators exerted divergent effects on the immunogenicity of CVA6, CVA10 and CVA16, with BPL exhibiting superior immune response induction for CVA10 and CVA16. Consequently, the evaluation of inactivators in preclinical evaluation is of paramount importance. Besides, there were two CVA6 vaccines inactivated by heating at 56°C for 30 minutes (**Table 1**), which is actually not practical in the vaccine scale-up process.

### 4.2 Live attenuated vaccines

The development of live attenuated vaccines (LAVs) is contingent on the screening of highly attenuated and genetically stable strains. Currently, research on live attenuated vaccines is at the stage of screening low-toxicity strains. Wang et al. constructed two mutant strains, CVA6-G64S and CVA6-G64T, by genetically engineering the 64th glycine position (G64) in the RNA-dependent RNA polymerase (3D polymerase) (64). These mutant strains exhibited a significantly reduced pathogenicity compared to the wild type, and the mutation frequency was significantly lower than that of the wild type under the mutagenic effect of ribavirin, suggesting that their replication fidelity was higher. This provides a theoretical basis for the use of CVA6 LAVs. However, the study did not include an immunogenicity assessment. Furthermore, it only examined the effect of mutations at a single locus without comparing the effect of mutations at other loci. In the future, further screening of highly attenuated vaccine candidates is needed to elucidate the key pathogenesis and to investigate the safety and immunogenicity of highly attenuated strains.

Despite the fact that endeavors to develop LAV of CVA6 have been extremely limited, LAVs have played a pivotal role in the prevention of viral infections, including smallpox, polio, and measles. Conventional LAVs are time-consuming involving the adaptation of virulent viruses to novel hosts, cell cultures, or suboptimal environments, resulting in a reduction in pathogenicity while retaining immunogenicity (65). Currently, genome editing, particularly CRISPR-Cas9, revolutionizes vaccine development by enabling precise modifications of pathogen genomes, leading to enhanced vaccine efficacy and safety (66). It has been posited that gene editing in inactivated vaccines might be a viable approach that merits further exploration.

### 4.3 Virus-like particle vaccines

Virus-like particle (VLP) vaccines employ the protein capsid of a virus devoid of genetic material, rendering it unable to infect but still capable of stimulating the human immune system to produce antibodies (67). Due to its unique advantages, such as its resemblance to natural virus particles and absence of viral genes, VLP has shown great promise in vaccine development with a high safety profile. The utilization of VLP in diverse research domains, including picornavirus vaccine development, has resulted in substantial advancements, as evidenced by notable breakthroughs in the field (68). Several studies have demonstrated the efficacy of CVA6 VLP vaccines in providing protection in mouse models (**Table 1**).

Production platforms for VLP vaccines encompass bacterial, yeast, baculovirus/insect cell (B/IC) expression systems, among others. Generally, co-expression of the *P1* and *3CD* genes is the main pathway to produce VLP (46). Bacterial and yeast platforms are suitable for simple and high-yield non-enveloped VLPs, B/IC platforms are suitable for complex and high-yield VLPs, mammalian cell platforms are best suited for complex enveloped VLPs, and plant platforms are suitable for low-cost VLPs (69). Existing studies have shown that CVA6 VLPs can be readily expressed in the B/IC expression system (59), *Picrosporum (* 60), and Chinese hamster ovary (CHO) cells (70). However, these studies have lacked a comparison of the production efficiency of different systems and the quality of the products, and it is therefore recommended that future studies compare those produced by different expression systems.

In order to conduct a more in-depth investigation into the factors that affect the production efficiency of CVA6 VLPs, and to improve this efficiency, Xing et al. used CHO cells to produce recombinant CVA6 virus-like particles and constructed the first kinetic model for the vaccine (70). This model is distinct from traditional recombinant protein production models in that it takes cell lysis into account in order to evaluate its effect on VLP release. This model has been shown to address the challenge of monitoring VLP release patterns, thus providing a more reliable tool for the development of CVA6 VLP vaccines.

It has been demonstrated that, due to the properties of the viral structure, CVA6 VLPs exhibit certain defects. A recent study by Kuijpers and colleague found that these defects manifest in two ways (71): first, the mechanical stability of CVA6 VLPs is poor, as evidenced by nanoindentation experiments which indicated that the force leading to the rupture of the viral capsid of CVA6 VLPs was significantly lower than that of mature virus particles. Second, CVA6 VLPs have RNA fragments encoding the viral protein, which increases their infectious potential. Addressing these shortcomings is crucial for enhancing the vaccine manufacturing process to ensure the stability and safety of the vaccine.

### 4.4 Subunit vaccines

Subunit vaccines consist of a portion of the immunogenic protein component of a vaccine virus that has been purified to act as an antigen and usually needs to be mixed with an immune adjuvant to enhance efficacy (72). The diversity of enterovirus serotypes increases the complexity of vaccine design, and the selection of highly conserved epitopes for subunit vaccine design can avoid the challenges of vaccine development posed by the high mutation rate of different enterovirus RNAs and enterovirus recombination between strains. Deng et al. developed a multivalent vaccine comprising EV-A71, CV-A16, CV-A10, CV-A6 and CV-B3, based on an immunoinformatics screen for antigenic epitopes exhibiting high conservatism and immunogenicity (53). Initially, a number of non-toxic and highly conserved antigenic epitopes in B cells, helper T lymphocyte (HTL) and cytotoxic T-cell lymphocyte (CTL) cells were predicted by computer. Thereafter, highly similar antigenic epitopes in the aforementioned five serotypes were screened out, and the antigenic epitopes were ligated by the 50 s ribosomal protein L7/L12 (rpIL) adjuvant to enhance the immunogenicity of the vaccine, and then DNA synthesis was carried out based on the protein sequences, and the expression plasmid was cloned and transformed into *Escherichia coli* to produce the vaccine protein (53). However, the results of animal experiments revealed that the antibodies produced by this vaccine exhibited a more significant neutralization ability against EV-A71 and CV-B3, and a non-significant neutralization ability against CVA16, with no neutralization ability observed against CVA6 and CVA10 (**Supplementary Table 1**). The suboptimal outcomes might be caused by low level cross-reactivity of key epitopes among these serotypes. It is noteworthy that no experiments were conducted to verify the vaccine’s protective effect. Furthermore, disparities in the spatial configuration of the proteins comprising each antigenic epitope following expression in *E. coli* may contribute to the suboptimal immunization response. The paucity of data on subunit vaccines underscores the necessity for further studies to elucidate the spatial configuration of expressed proteins and the role of B-cell antigenic epitopes as well.

The subunit approach is associated with a reduction in immune response, necessitating the incorporation of immunoadjuvants to enhance immune stimulation. Beyond that, optimization of delivery systems, tuning the size of particulate vaccines, targeting specific cells (e.g., dendritic cells) of the immune system, and adding components to aid vaccine efficacy in whole immunized populations (e.g., promiscuous T-helper epitopes) require harmonization (73). Nevertheless, subunit vaccines present the immune system harmless fragments of the target pathogen, to trigger humoral and cellular immune activation (74). It is of great value in the development of monovalent as well as multivalent vaccines of enteroviruses in the future.

### 4.5 DNA vaccines

A DNA vaccine is a genetically engineered vaccine that contains genes encoding specific antigens and sequences to initiate and terminate gene expression (75). Following injection into the body, the DNA vaccine enters the cell and continuously synthesizes the corresponding antigenic proteins within the cell, producing a long-lasting immune effect (49). A further advantage of DNA vaccines is that they are free of infectious particles and infectious RNAs, which do not cause viral infections and provide a high level of safety (76). Bello et al. developed a DNA vaccine comprising a DNA sequence that contains the entire VP1 protein of EV-A71 and six known neutralizing B-cell epitopes from EV-A71, CVA16, CVA10 and CVA6 for the synthesis of VP1me, and subsequently the VP1me gene was cloned into the mammalian expression vector pVAX1 to produce a VP1me gene vaccine (49). Subsequent immunofluorescence staining and immunoblotting experiments demonstrated that the DNA vaccine could induce HEK293A cells to express VP1me protein. BALB/c mice were immunized with the DNA vaccine, and the serum levels of IgG antibodies against VP1me protein and the frequency of CD8+ T cells in splenocytes were examined. The results demonstrated that the DNA vaccine exhibited superior immunogenicity compared to the control group, as indicated by the elevated levels of both humoral and cellular immunity. However, the study did not ascertain whether the antibodies produced by the vaccine possessed a neutralizing effect on the target virus. As the data DNA vaccine of CVA6 is limited, its immunogenicity, efficiency, humoral and cellular immunity, safety needs more critical and scientifically-designed *in vivo* evaluation in the future.

### 4.6 mRNA vaccines

The mRNA vaccines, akin to DNA vaccines, are vaccines in which mRNA fragments encoding viral antigens are injected into the body via a delivery system to enable human cells to synthesize antigens autonomously, thereby activating the immune system to generate an immune response against the pathogen (77). In comparison with DNA vaccines, mRNA can directly enter the cytoplasm for translation and can produce antigens to activate the immune response more expeditiously (78). As a new generation technology, mRNA vaccines demonstrate significant advantages over traditional vaccines, including enhanced safety, efficiency in production, and immunogenicity (79). These advantages have led to the identification of broad application prospects for mRNA vaccines. The first successful application of an mRNA vaccine in the prevention of SARS-Cov-2 has garnered considerable attention and recognition (80). In recent years, there has been a significant increase in the number of mRNA vaccine studies, with current research focusing on mRNA vaccines for significant viruses, including the influenza virus, respiratory syncytial virus, and HIV (81–84). With regard to delivery systems, the development of lipid nanoparticle (LNP) technology has led to significant advancements in the stability and targeting of mRNA (82).

A team from China recently conducted a study in which they evaluated the D3a CVA6 mRNA vaccine at the mouse level for the first time (46). The backbone sequence containing 5′UTR, *Spe*I cleavage site, Kozak sequence, tPA signal peptide, humanized *P1* region (or *VP1* region), *BamH*I cleavage site, 3′UTR, and 120 adenylic acids (ployA) was constructed for *in vitro* transcription. They compared the immunogenicity and protective effects of the D3a CVA6 mRNA vaccine with the VLP vaccine produced in *pichia pastoris*. The study investigated the protective effects of VLP and core-shell structured lipopolyplex mRNA (LPP-mRNA) vaccines against CVA6. Their results suggested that cellular immunity appeared first and protected the animals from lethal doses. The VLP vaccine was found to elicit neutralizing antibodies and enhance cellular immunity, thereby protecting mice against a lethal CVA6 challenge. In contrast, the LPP-mRNA vaccine induced robust T-cell immunity, providing cross-protection against CVA10. This study represents the first trial of a CVA6 mRNA vaccine and the first comparison of VLP and mRNA vaccine immunogenicity and protective effects based on the D3a CVA6 sub-genotype. The findings of this study offer valuable insights for the development and immunization strategies of EV vaccines.

Effective virus-specific cellular response is essential for viral clearance, as the emerging variants have been demonstrated to efficiently evade prior humoral immunity (85). Recently, Tai et al. developed a mRNA-based T-cell-inducing antigen that encodes 3 SARS-CoV-2 peptides enriching human HLA-I epitopes, which induced broad and potent cellular responses in both humanized HLA-transgenic mice and nonhuman primates. This mRNA format allows for *in situ* production of 3 epitope-enriched peptides in tandem (HLA-EPs), which contributes to the rapid formation of the immunopeptidome and efficient access to the HLA compartment of antigen-presenting cells (86). The findings of this study suggest that the development of vaccines using antigen designs that target the cellular immune response, or the combinational activation of the humoral and cellular immune responses, may represent a promising strategy for the creation of next-generation vaccines. Indeed, a significant benefit of mRNA-based vaccines is the endogenous synthesis of encoded proteins. This process enables the presentation of foreign antigens by the major histocompatibility complex (MHC) (87).

### 4.7 Multivalent vaccines

The current multivalent vaccines that include serotypes such as CVA6 principally comprise inactivated vaccines, VLP vaccines, subunit vaccines, and DNA vaccines (**Table 1**). Among these, inactivated and VLP vaccines have been the subject of substantial research by various research teams, while the evidence for subunit and DNA vaccines remains comparatively limited. All the tetravalent vaccines listed in **Table 1** encompass four serotypes, namely CVA6, CVA10, CVA16, and EV-A71. However, due to the varying immunogenicity of antigens across serotypes and the differing concentrations of neutralizing antibodies (NtAb) produced by these antigens, there is a possibility of one immunogen predominating over others, resulting in an imbalanced immune response and inadequate protection against the target pathogen(s). Furthermore, due to antigenic interference among different antigens, the concentration of NtAb produced by multivalent inactivated vaccines may not match that of monovalent vaccines. Consequently, to ensure the production of sufficient NtAb and to induce a balanced immune response for each serotype, the ratio of the different antigens in multivalent vaccines must be optimized (55, 56).

In a study of a tetravalent VLP vaccine against EV-A71, CVA16, CVA10, and CVA6, the tetravalent VLP vaccine exhibited NtAb titers similar to those of the monovalent vaccine, and the binding capacity of sera from mice in the tetravalent VLP vaccine was comparable to that of the monovalent vaccine (54). These findings suggest that the antigenic components of the four antigens are well compatible in the tetravalent vaccine. The study also observed that the NtAb titers of CVA10, CVA6 and CVA16 monovalent VLP were significantly lower than those of EV-A71 VLP, suggesting that CVA6, CVA10 and CVA16 antigens may possess reduced immunogenicity compared to those of EV-A71, and that increasing the proportions of these antigens in a multivalent vaccine could potentially bridge the immunogenicity gap with EV-A71 (54). These results indicate in intra- and inter-vaccine interference among components can lead to suboptimal responses given the complexity of immune responses to multiple antigens (88, 89). This phenomenon has the potential to influence the overall immune response, thereby reducing the effectiveness of the vaccine. The mechanisms underlying this interference may include competition for T-cell help and B-cell activation, which are crucial for generating a strong and lasting immune response (90). In vaccine responses, T cell-B cell interactions are orchestrated through T follicular helper (Tfh) cells, which promote germinal center formation and B cell maturation. Antigen-presenting dendritic cells prime CD4+ T cells to differentiate into Tfh cells, which secrete IL-21 and express CD40L to drive B cell proliferation, somatic hypermutation, and antibody class-switching. This process generates high-affinity, long-lived plasma cells and memory B cells, critical for durable immunity (91). Adjuvants enhance these interactions by boosting dendritic cell activation and antigen presentation. Dysfunctional Tfh-B cell crosstalk can impair vaccine efficacy (92). Targeting these pathways optimizes vaccine design, particularly for pathogens requiring robust neutralizing antibodies. Taken together, the immunological perspective on antigenic interference in multivalent vaccines highlights the intricacies involved in the formulation of effective vaccines.

Antigenic interference, in which co-administered antigens compete for immune resources, can be studied via *in vitro* B/T-cell co-cultures with multiplexed antigen exposure or *in vivo* models tracking germinal center dynamics (e.g., single-cell RNA-seq). Mitigation strategies have been developed to address these challenges, including the optimization of antigen dosing ratios to prevent dominance, the use of adjuvants (e.g., TLR agonists) to broaden APC activation, and the implementation of staggered immunization schedules to reduce competition (93). Preclinical studies demonstrate that the strategic pairing of adjuvants with antigens, in conjunction with the temporal modulation of prime-boost intervals, fosters epitope spreading without inducing immune overload. This approach achieves a balanced immunogenicity profile across multivalent vaccines (94).

## 5 Strain screening and immunogenicity

Vaccine strain screening is a key step in preclinical research and it is important to screen strains with high antigenicity and stability. Among the KMB17 cell-adapted strains, only KYN-A1205 caused sickness or partial death in suckling mice, and its virulence was greater than that of the RD cell-adapted strain (95). The KYN-A1205 strain caused severe sensitization of mouse muscle tissue and pathological changes, including muscle necrosis and nuclear fragmentation in the forelimb and hindlimb. It showed strong pathogenicity, good immunogenicity and genetic stability, making it suitable for use as an experimental CVA6 vaccine candidate (95). Antigenic analysis showed cross-antigenicity between strains of CVA6 subgenotypes A, B2 and D1-D3, meaning that antibodies raised against one subtype cross-neutralize other subtypes of CVA6 (96).

Due to differences in immunogenicity and reactivity of different strains, the use of different strains in the NtAb CVA6 assay may affect NtAb titers. Gao et al. selected S112 as a test strain to compare the titer of different NtAb and their cross-neutralization ability with other strains (97). S112 is easy to neutralize with the lowest MAX/MIN ratio, making it an ideal choice for assessing the immunogenicity of the CVA6 vaccine and broad potency assessment (97). In addition, antibodies against strain S112 were found to have good broad-spectrum cross-protection against genotype A and subtypes D1 and D3, suggesting that S112 could be a candidate strain for CVA6 vaccine.

To standardize immunogenicity assessment criteria for vaccines and to ensure the quality and validity of antibody concentration for immunogenicity assessment, the National Institutes for Food and Drug Control (NIFDC) has established the first national standard for neutralizing antibodies against CVA6, in which the candidate has good long-term stability with 3-year follow-up of NtAb titers (98). The establishment of harmonized antibody concentration standards can effectively reduce inter-laboratory and strain testing variation and improve the accuracy of vaccine evaluation.

## 6 Preclinical *in vivo* evaluation

### 6.1 Mouse model

Currently, the EV vaccine evaluations have been mainly conducted in BALB/c or Institute of Cancer Research (ICR) mouse models (**Figure 1**). In general, intraperitoneal (i.p.), intramuscular (i.m.), and intracranial (i.c.) injections are the traditional routes of infection (48). However, the natural routes of infection for EVs are gastrointestinal and respiratory. Therefore, it is important to construct animal models of the natural routes of infection for vaccine evaluation. Li et al. constructed an orally infected 10-day-old ICR mouse model to mimic the normal routes of infection (50). The model was able to mimic the typical symptoms and pathological changes of infection through physical injury caused by gavage, showing CVA6 skin symptoms such as, as well as skin hair loss, neurological complications such as poor mental health, lethargy, panic, ataxia and limb paralysis (99). In addition, CVA6 replication and CVA6 VP1 antigen were detected in the brain and spinal cord tissue of CVA6-infected mice, suggesting that the spinal cord may be the pathway for the virus to cross the blood-brain barrier (50).

Sun et al. further confirmed the inflammatory damage caused by CVA6 in the central nervous system (CNS) of neonatal mice, with CVA6 preferentially infecting astrocytes and neurophilic CVA6, and found that CVA6 viral antigen was co-localized with the astrocyte marker (99). The co-localization of CVA6 viral antigen and GFAP suggests that astrocytes may be the primary infected cells in the context of CVA6 infection. The results of this study showed that CVA6 infection resulted in pathological changes, including edema and swelling of neuronal cells in mouse brain tissue. This suggests that neurons may also be damaged, leading to neurological dysfunction, paralysis and other symptoms.

### 6.2 Non-human primate model

The immune system of neonatal mice has not yet developed, which poses a significant challenge in inducing a complete and fully functional immune response (100). In addition, mouse models of HFMD are typically infected a few days after birth and the age of infection differs significantly from that of human infants, making it difficult to fully simulate the manifestations observed in certain age groups. In addition, the physiology and immune functions of mice differ significantly from those of humans (101), and mouse models are only able to mimic one or more clinical symptoms of HFMD, but cannot fully reproduce the pathogenic characteristics and pathogenesis of HFMD infection. In order to obtain infection data that more closely resembles the human infection situation, Duan et al. successfully established the first NHP model of CVA6 infection. Rhesus monkeys have a high degree of genetic, physiological and immune system similarity to humans and are able to display symptoms of HFMD analogous to those seen in humans (102). This allows the rhesus monkey model to better mimic the course and progression of infection in humans. They used CVA6 to infect 3–4 months old rhesus monkeys via the respiratory or gastrointestinal tract. Infected rhesus monkeys exhibited symptoms similar to patients, including fever, skin rashes or herpes-like lesions, blood cell changes, viremia and virus shedding (102). Pathological observations show an inflammatory response in the intestinal tract and lymph node tissue. During the recovery period, acute symptoms subsided, but viral replication and shedding persisted, high levels of NtAb continued to be produced, and there were no significant differences in the outcome of infection whether the monkeys were infected via the respiratory or gastrointestinal tract. This model provides an important tool for studying the pathogenic mechanism and immune response in primates, and facilitates the transition of relevant vaccine and drug studies to clinical trials.

NHP models are critical in vaccine development due to their genetic, immunological, and physiological proximity to humans. They enable rigorous evaluation of vaccine safety, immunogenicity, and protective efficacy in complex immune systems, bridging preclinical and clinical trials. NHPs recapitulate human-like immune responses to adjuvants, dosing regimens, and mucosal or systemic delivery routes, informing optimal vaccine design (103). For example, NHP studies validated COVID-19 vaccine correlates of protection (104). However, ethical constraints, interspecies variability, and high costs necessitate careful experimental design. Advanced imaging, multi-omics profiling, and controlled challenge studies in NHPs remain indispensable for de-risking clinical translation.

### 6.3 Preclinical animal model selection

The development of enterovirus vaccines relies on animal models to evaluate immunogenicity, protective efficacy, and safety. The following criteria are considered essential in the selection of ideal models:

1. Susceptibility: The organism exhibits the capacity to replicate natural human infection pathways, including the oral and enteric routes, while concurrently manifesting clinical indications characteristic of various pathological conditions, such as neurological or cardiac diseases. Transgenic mice that express human receptors (e.g., hSCARB2 for EV-A71) facilitate the study of pathogenicity (105).
2. Immune response alignment: It is imperative that models generate human-like humoral and cellular immunity (106). Neonatal mice, despite their immunological immaturity, are utilized in conjunction with adjuvants to amplify response (107).
3. Scalability: Mice offer cost-effective, high-throughput screening due to two factors: their rapid breeding rate and their moderate susceptibility (108).
4. Translational relevance: NHP have been employed as a model system to validate cross-species protection, yet they are subject to ethical and cost constraints. In order to optimize the clinical translation of NHP studies, it is essential that these studies employ controlled challenge protocols, longitudinal immune monitoring (neutralizing antibodies, cellular responses), and dose-ranging designs that mirror those employed in human trials. Co-administration studies and mucosal sampling have been shown to refine delivery strategies (109). NHP-derived correlates of protection (e.g., antibody titers) directly inform clinical endpoints, while safety and toxicity data guide risk mitigation in phase I trials. The implementation of rigorous statistical powering and heterologous prime-boost testing in NHPs has been demonstrated to reduce the incidence of late-stage clinical failure risks (110, 111).

## 7 Comparison of vaccine platforms: efficacy, safety, stability, cost, and production

**Figure 5:**
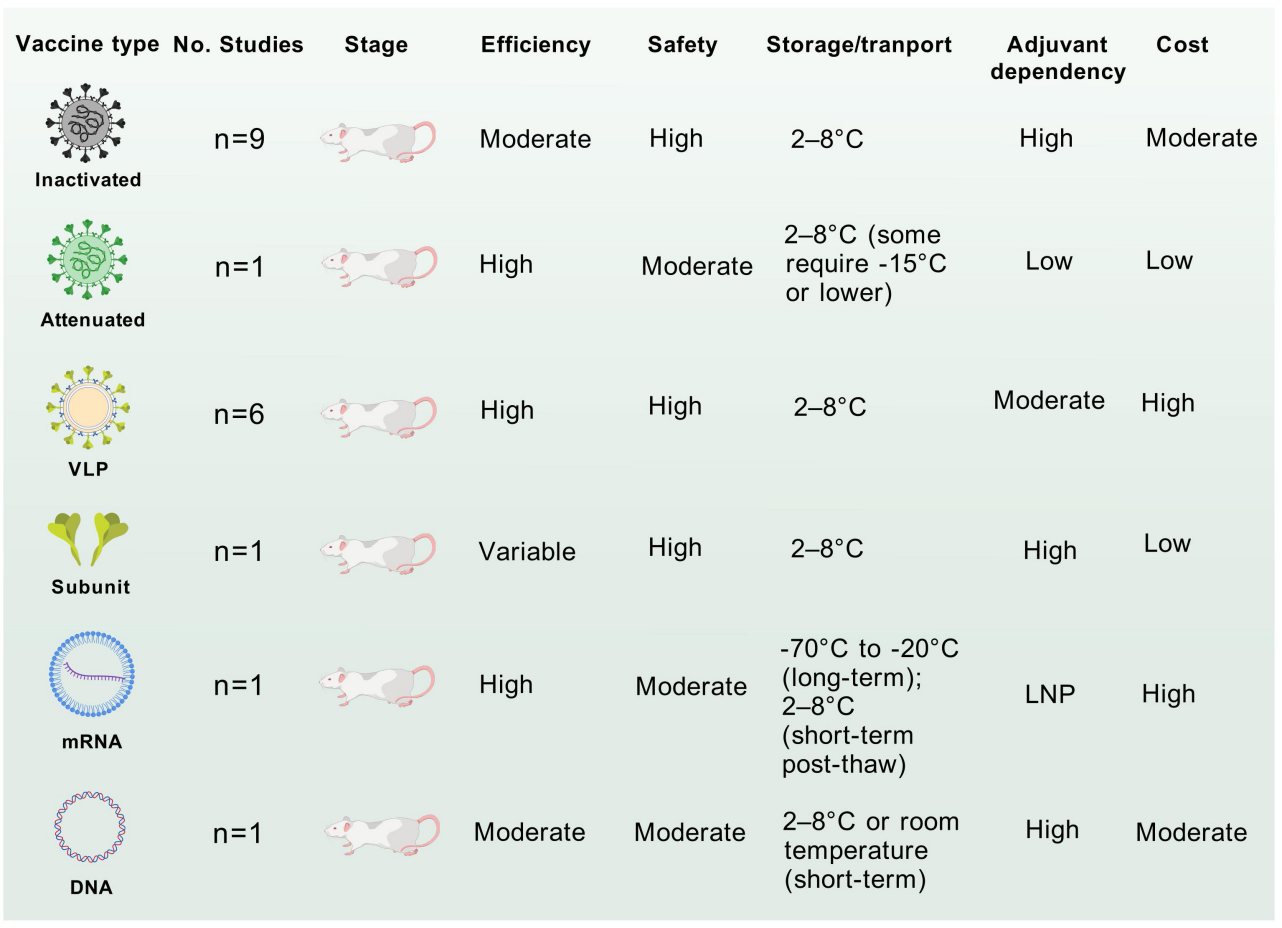
Comparative analysis of CVA6 vaccines of different technology. The application potential and safety for each vaccine were comprehensive assessed by the accumulating evidence. These discourses, however, do not necessarily represent the final path to clinical translation of CVA6 vaccine.

Inactivated vaccines, such as those targeting polio and enterovirus EV-A71, demonstrate moderate efficacy, often necessitating adjuvants or booster doses to enhance immunogenicity (42). Their non-replicative nature ensures high safety, while stability at 2–8°C facilitates distribution in resource-limited settings (**Figure 5**). However, production costs remain moderate due to requirements for pathogen culture and inactivation. In contrast, live-attenuated vaccines elicit robust and durable immunity by mimicking natural infection but can also elicit unintended off-target effects (112). Despite cold chain dependency, their low production cost supports widespread use (**Figure 5**). VLP vaccines achieve high efficacy through structural mimicry of native virions while maintaining excellent safety profiles due to the absence of genetic material (**Figure 5**) (69). However, their reliance on recombinant protein assembly elevates production costs, and refrigeration is required to prevent aggregation.

DNA vaccines induce strong cellular immunity (113). Their room-temperature stability and scalable plasmid-based production offer logistical advantages (**Figure 5**), yet limited clinical adoption underscores unresolved delivery challenge (114). mRNA vaccines, notably employed during the COVID-19 pandemic, combine high efficacy with rapid design flexibility (80, 115). Transient reactogenicity linked to lipid nanoparticle (LNP) components and stringent ultracold storage requirements (-20°C to -70°C) pose challenges, though costs are moderated by cell-free *in vitro* transcription platforms(**Figure 5**) (79). Subunit vaccines, such as hepatitis B vaccines, exhibit variable efficacy dependent on adjuvants but are highly safe due to purified antigen formulations (116). Their stability at 2–8°C and low-cost production via yeast or bacterial expression systems make them logistically favorable.

Taken together, mRNA and VLP technologies prioritize rapid development and high efficacy but face stability and cost barriers, whereas inactivated and subunit vaccines provide pragmatic solutions for global deployment, particularly in resource-constrained regions.

## 8 Potential adverse effects and safety concern

The potential adverse effects, toxicity concerns, and human risks associated with vaccines have garnered increasing attention in recent years, particularly in the context of the COVID-19 pandemic, when the mRNA vaccines were first applied. While mRNA vaccines have been heralded for their rapid development and efficacy, understanding their safety profile is crucial for public health. Two major concerns of mRNA vaccines include the potential for antibody-dependent enhancement (ADE) (117) and systemic leakage of vaccine components that might lead to unintended immune responses (115). Moreover, the reactogenicity of mRNA vaccines has been a focal point in clinical assessments as varying levels of reactogenicity that could influence public perception and vaccine uptake (118). VLP-based vaccines are generally safe, while novel adjuvants may enhance reactogenicity, necessitating long-term monitoring (119).

Inactivated vaccines, such as the EV-A71 vaccine targeting severe HFMD, demonstrate robust safety profiles with primarily mild local (pain, erythema) reactions or low-grade fever, as evidenced by phase III trials (42). Live-attenuated vaccines can stimulate both humoral and cellular responses, while adverse events such as headache, muscle pain, fever diarrhea, bloody stool, and vomiting need critical evaluation in clinical trial, particularly among children (120, 121). DNA vaccines pose theoretical concerns about genomic integration, with transient injection-site reactions as the main side effect (122). Subunit vaccines are highly safe, though adjuvants like aluminum salts may trigger local inflammation or rare hypersensitivity (123, 124). Collectively, manufacturing rigor and post-marketing surveillance are critical for all platforms to mitigate residual risks. Beyond that, challenges are also faced for vaccine stability associated with storage conditions (125). Vaccine stability is influenced by thermal degradation, pH fluctuations, hydrolytic damage, and UV degradation. Excipient formulation (e.g., stabilizers, buffers) and storage conditions (humidity, temperature) critically impact macromolecular integrity. Manufacturing stresses (shear forces, freeze-thaw cycles) may destabilize antigen conformation or LNPs in mRNA vaccines. However, Muramatsu, et al. demonstrate that mRNA-LNPs can be lyophilized (freeze-dried) and stored at ambient temperature for 12 weeks and at 4°C for 24 weeks without substantial changes to their physical properties or mRNA delivery efficiency (126). Their findings on the long-term storage and stability of lyophilized mRNA-LNPs are critical to the widespread development and implementation of LNPs for COVID-19 and other diseases.

## 9 Future prospects for CVA6 vaccine development and clinical translation

**Figure 6:**
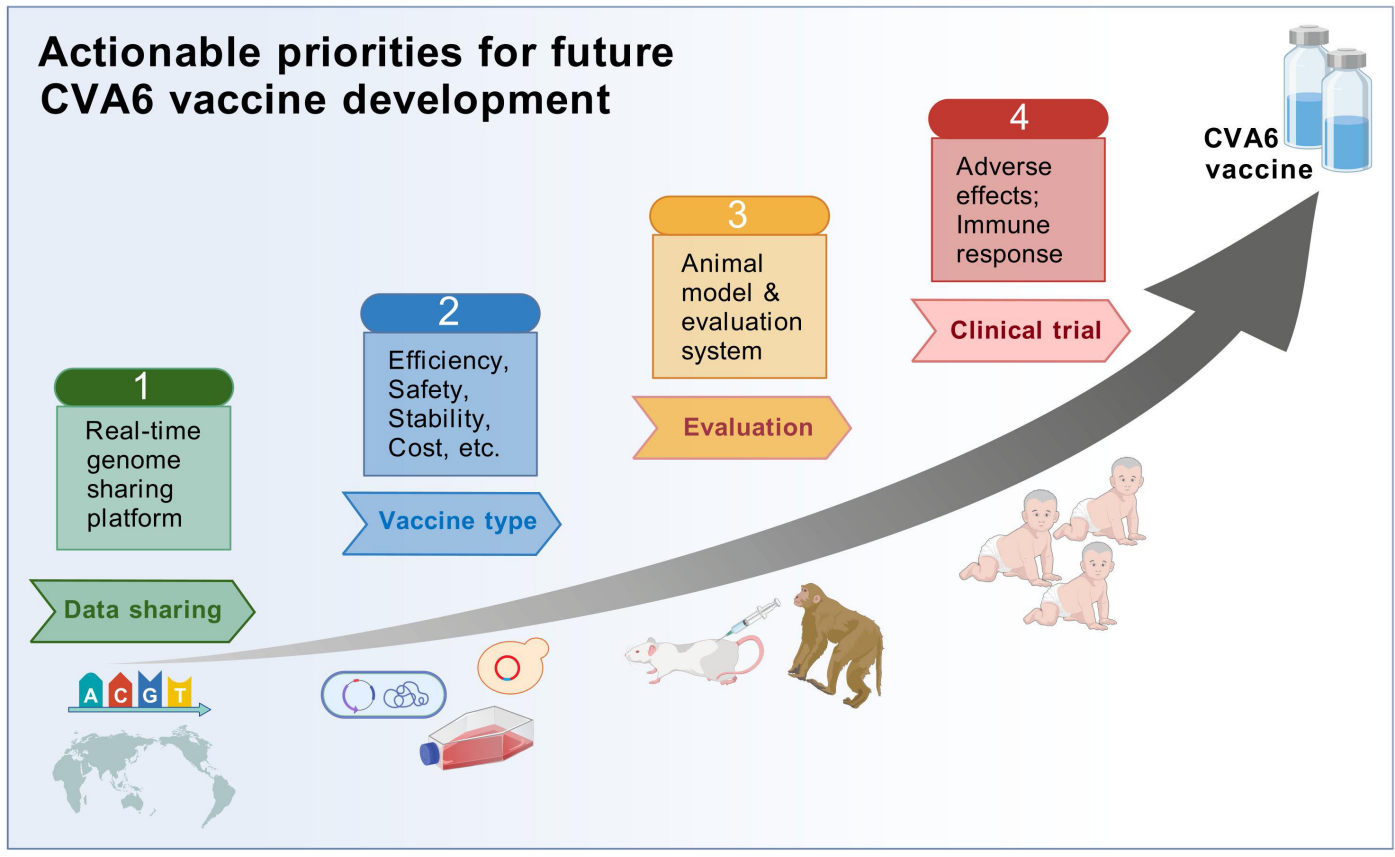
Roadmap for actionable priorities for future CVA6 vaccine development. International collaborative efforts should be focused on global genome data sharing, comprehensive vaccine platform comparison, efficient animal models and scientific clinical trials.

With the ongoing global epidemic of CVA6 and the public health challenges it poses, vaccine research has become a key link in building the HFMD prevention and control system. Based on current research progress and technological breakthroughs, the future development of CVA6 vaccines must focus on 1) multivalent vaccine development and potential antigenic interference among components; 2) the use of next-generation vaccine technologies such as mRNA technology; 3) innovative vaccine production technology; 4) scientific preclinical experiments and clinical trials; 5) conflict of antigenic variation and broad-spectrum immune protection (**Figure 6**).

In 2016, the Chinese FDA-approved EV-A71 vaccine was first introduced in China and then programmed as a category 2 vaccine (44). Li et al. evaluated the immunogenicity and safety of three inactivated enterovirus A71 vaccines in children aged 6–35 months in China and found high seroconversion rates, but no serious adverse events in any group (42). Over the past decade, several regional studies have also shown that EV-A71 vaccination significantly reduced the incidence of EV-A71-associated HFMD in China. The latest real-world research data showed that EV-A71 vaccine effectively controlled the occurrence of EV-A71 HFMD in China, but it varied due to differences in vaccination coverage and population density, and >20% vaccination coverage was recommended for children under 5 years of age (43). Therefore, the control of enteroviruses depends not only on the success of vaccine development, but more importantly on whether the vaccination coverage of the age-appropriate population can meet the standard. For example, He et al. found that EV-A71 vaccination in Nanchang, China, consistently maintained >20% coverage, and HFMD pathogen surveillance showed that no EV-A71 HFMD cases were monitored in the area after 2018. The results of this study are consistent with the findings of the real-world studies mentioned above, suggesting that effective vaccination rates are essential to prevent rebound of viral infections. At present, CVA6 vaccines of all types are still in preclinical development, and the majority of studies have centered on inactivated and VLP vaccines, both of which have exhibited superior immunogenicity and protection efficiency (**Figure 5**). Furthermore, the successful experience with inactivated EV-A71 vaccines in China serves as a valuable template for the development of inactivated CVA6 vaccine.

Although CVA6 started to circulate dominantly in China and many other countries, pathogen spectrum shift occurs periodically, which is a challenge for vaccine development (6, 7, 32, 127). It is recommended that the future focus of research and development should be on multivalent vaccine or mRNA vaccine. A concerted effort is required to achieve a harmonization between antigenic variation and the overarching objective of attaining comprehensive immune protection. The concepts underpinning the design of the SARS-Cov-2 mRNA vaccine constitute a significant reference point for the ongoing development of future enterovirus mRNA vaccines. Furthermore, the combination of mRNA vaccines with other antigenic vaccines has been demonstrated to be effective in the induction of both cellular and humoral immunity (86). However, due to the short market and application time of mRNA vaccines in humans, the safety evaluation of mRNA vaccines requires longer and continuous monitoring.

Finally, a multitude of challenges must be surmounted to achieve clinical translation, largely due to the rigorous regulatory frameworks encompassing preclinical safety (toxicology, immunogenicity), chemistry, manufacturing, and controls (CMC), and phased clinical trials (Phase I-III). It is imperative to note that critical steps include Investigational New Drug (IND) submissions, Good Manufacturing Practice (GMP) compliance, and protocol alignment with International Conference on Harmonization (ICH) guidelines. Risk management plans address safety uncertainties, while real-time stability data ensure product integrity. Subsequent to approval, Phase IV surveillance monitors long-term efficacy and adverse events. The implementation of global harmonization measures, such as those facilitated by the World Health Organization (WHO) prequalification program, has been demonstrated to expedite the deployment of countermeasures, a phenomenon that is particularly salient in the context of pandemic response.
