# Supplementary material for: Recent advances on coxsackievirus A6 vaccine research
Source: Front Immunol. 2025 Jun 6;16:1603028. doi: 10.3389/fimmu.2025.1603028 (PMC12179169; doi:10.3389/fimmu.2025.1603028)
Supplement: Supplementary file 1 [file Table1.docx]

**Supplementary table 1. Immunogenicity and efficiency of different vaccine platforms**

| **Year** | **Vaccine type** | **Serotype(s)** | **Animal model** | **Protection rate** | **Immunogenicity** | **Cross-protection** |
| --- | --- | --- | --- | --- | --- | --- |
| 2025 | VLP, mRNA | CVA6 | ICR mice | VLP: 100%  mRNA: robust T-cell immunity | Anti-VLP sera  IgG titers>1:1000 in BALB/c mice and 1:10000 in ICR mice; | mRNA vaccine of CVA6 protected mice against a lethal dose of CVA10 |
| 2025 | VLP | CVA6 | BALB/c mice | 100%, n = 5 | The geometric mean titer (GMT) against CVA6-TW-00141 prepared on Vero-KRM1_#11 cells was from 256 to 1024;  The GMT against other CVA6 strains were from 64 to 1024 | NA |
| 2024 | VLP | CVA6 | Neonatal ICR mice | 100%, n=6 | The average neutralization dilution ratios of anti-D3a sera were 1:16,1:32, and1:512;  The average neutralization dilution ratios of anti-D3b sera were 1:2, 1:32, and1:128 respectively. | 100% protection rate against D3a and D3b challenge |
| 2024 | DNA | CVA6, CVA10, CVA16, EV-A71 | BALB/cAJcl mice | NA | IgM: 1:3200, IgG: 1:3200  NtAb were induced in suckling mice. | NA |
| 2022 | Inactivated | CVA6 | ICR mice | 100% | NA | NA |
| 2021 | Inactivated | CVA6 | Kunming mice | 100%, n=20 | Dose 1: 1.5 µg, NtAb titer: 1230  Dose 2: 4.5 µg, NtAb titer: 911 | NA |
| 2021 | Inactivated | CVA6 | BALB/c mice | 100%, n=10 | NA | NA |
| 2020 | Sub-unit | CVA6, CVA10, CVA16, CVB3, EV-A71 | Mice | NA | NtAb titer against EV-A71: 140;  NtAb titer against CV-B3:10;  NtAb titer against CV-A16:8 | NA |
| 2018 | VLP | CVA6, CVA10, CVA16, EV-A71 | BALB/c mice  ICR mice | 92%, n=12 | Tetra-VLP group strongly neutralized  EV71/G082 with GMTs of 1825;  CVA16/SZ05 with GMTs of 4598;  CVA10/ S0273b with GMTs of 362;  CVA6/Gdula with GMTs of 228 | NA |
| 2018 | Inactivated | CVA6, CVA10,  CVA16 | BALB/c mice | CVA6: 96.2%, n=10; CVA10: 100%, n=10; CVA16: 59.1%, n= 10 | BPL-inactivated trivalent vaccine induced high-titer antigen-specific IgG against CVA6, CVA10 or CVA16;  Higher NtAb of CVA6 than CVA10 and CVA16. | NA |
| 2018 | Inactivated | CVA6, CVA10 | BALB/c mice, neonatal mice | Vaccine protection: 80%, n=10; neonatal passive immunization 100%, n=10 | The antibody GMTs for CVA6 and CVA10 monovalent vaccine-immunized antisera were 1365 and 1195, respectively;  The GMT was up to 1024 in the CVA6/CVA10 bivalent vaccine-immunized mice. | NA |
| 2017 | Inactivated | CVA6 | ICR mice | 100%, n=10 | NA | The CVA6 antiserum did not neutralize EVA71 and CVB4 |
| 2016 | Inactivated | CVA6, CVA10, CVA16, EV-A71 | Mice, rabbits | NA | NtAb titer against EV-A71: 708;  NtAb titer against CV-A6: 100;  NtAb titer against CV-A10: 16  NtAb titer against CV-A16: 22 | CVA6 antisera neutralized CVA6 infections but did not cross-neutralize EV-A71, CVA10 or CVA16 infections |
| 2016 | VLP | CVA6 | Neonatal ICR mice, BALB/c mice | 100%, n=12 | GMT: 179,594 | VLP-immunized mice showed efficient cross protection against heterologous CA6/S0087b strain |
| 2016 | VLP | CVA6 | ICR mice | 100%, n=14 | GMT: 17,959 | NA |
| 2016 | Inactivated | CVA6 | BALB/c mice | 100% | Maternal immunization and showed 100% protection of neonatal mice from lethal CA6 challenge. | NA |
| 2015 | Inactivated | CVA6, CVA16,  EV-A71 | A129 mice, AG129 mice | EV-A71: 100%, n=6; CVA16: 100%, n=5; CVA6: 100%, n=6 | NtAb titer against EV-A71: 485;  NtAb titer against CV-A6: 1436;  NtAb titer against CV-A10: 285 | NA |

NA: not available
